# Methodological comparison of bronchoalveolar lavage fluid-based detection of respiratory pathogens in diagnosis of bacterium/fungus-associated pneumonia in critically ill patients

**DOI:** 10.3389/fpubh.2023.1168812

**Published:** 2023-05-15

**Authors:** Luwen Zhang, Fanbo Lu, Yuerong Wang, Juanjuan Ji, Yuanhong Xu, Ying Huang, Min Zhang, Moyan Li, Jinxing Xia, Bo Wang

**Affiliations:** ^1^Department of Clinical Laboratory, The First Affiliated Hospital of Anhui Medical University, Hefei, Anhui, China; ^2^Institute of Pediatrics, Children’s Hospital of Nanjing Medical University, Nanjing, Jiangsu, China; ^3^Department of Respiratory and Critical Care Medicine, The First Affiliated Hospital of Anhui Medical University, Hefei, Anhui, China

**Keywords:** bacterium/fungus-associated pneumonia, bronchoalveolar lavage fluid, PCR, methodological comparison, critically ill patients

## Abstract

**Background:**

Bacterium/fungus-associated pneumonia (BAP/FAP) is the prominent cause of high mortality and morbidity with important clinical impacts globally. Effective diagnostic methods and proper specimen types hopefully facilitate early diagnosis of pneumonia and prevent spread of drug-resistant bacteria/fungi among critically ill patients.

**Methods:**

In the present study, 342 bronchoalveolar lavage fluid (BALF) samples were collected from critically ill patients with pulmonary infections between November 2020 and March 2021. The BALF materials were comparatively employed to screen BAP/FAP through microscopy, culture, antigenic marker and PCR-based methods. The limit of detection (LOD) of cultures and PCR for bacteria/fungi was determined by serial dilution assays. Specimen slides were prepared with Gram staining for microscopic examinations. Microbial cultures and identifications underwent routine clinical protocols with the aid of mass spectrometry. (1,3)-β-D-glucan and galactomannan tests with BALF were carried out accordingly. Direct detection of pathogens in BALF was achieved through PCR, followed by sequencing and BLAST in GenBank database for pathogenic identification. The subjects’ demographic and clinical characteristics were well evaluated.

**Results:**

BAP/FAP was identified in approximately 47% of the subjects by the BALF-based PCR. The PCR-based diagnostic methods showed improved detection performance for fungi with good LOD, but performed similarly for bacteria, when compared to the cultures. There was poor agreement among traditional microscopy, culture and PCR assays for bacterial detections (kappa value, 0.184 to 0.277). For overall bacterial/fungal detections, the microscopy showed the lowest detecting rate, followed by the cultures, which displayed a slightly higher sensitivity than the microscopy did. The sensitivity of PCR was much higher than that of the other means of interest. However, the traditional cultures rather than antigenic marker-based approaches were moderately consistent with the PCR-based methods in fungal species identification, particularly for *Candida* and *Aspergillus* spp. Our findings further revealed that the age, length of hospital stay, invasive procedures and cerebral diseases were likely considered as main risk factors for BAP/FAP.

**Conclusion:**

Screening for BALF in critically ill patients with suspected pneumonia pertaining high risk factors using combined PCR-based molecular detection strategies would hopefully contribute to early diagnosis of BAP/FAP and improved prognosis of the patients.

## Introduction

Pneumonia is a common and acute respiratory infection that affects host alveoli and distal airways. It is now considered as the largest single threat to human health worldwide, surpassing cancers, heart diseases, AIDS, tuberculosis or malaria, and causes substantial morbidity and mortality globally (1–3).

The current incidence of pneumonia rises with age (4), lack of rapid and accurate diagnostic methods to identify targeted pathogens in the clinic has led to frequent and, in most cases, unnecessary empirical use of broad-spectrum antibiotics, a practice that has further resulted in current increase in drug resistance (5). The reported incidence rate of pneumonia varies geographically worldwide including China (6–8), its regional incidence in Northeast and East China was remarkably high (9). Therefore, rapid identification of causative agents of pulmonary infections help to initiate appropriate antimicrobial therapy, and ensure favorable treatments to control preventive infections (2, 10).

The microbiological diagnosis of pulmonary infections is fundamentally based on analysis of samples from host bronchial tree (11). The role of fiberoptic bronchoscopy (FOB) as an aid in the diagnosis of pneumonia has expanded in recent years. Various techniques, including bronchoalveolar lavage (BAL) *via* FOB, have been regarded as valuable and consistently accepted safe approaches in diagnosis of pneumonia (3). A review of 23 studies analyzing the diagnostic utilization of BALF in ventilator-associated pneumonia (VAP) reported mean values of sensitivity of 73 ± 18% and a specificity of 82 ± 19% (12). Additionally, BALF exhibited a higher sensitivity for Galactomannan (GM) tests compared to serum (13).

The most common method for diagnosis of pulmonary infections is microscopic examinations of Gram-stained sputum films. However, such diagnosis varies according to disease severity. Of note, microbiological diagnosis in pulmonary infections cannot be available in nearly 50% of such patients (14, 15). Sputum and blood sample cultures were primarily recommended in patients with pneumonia symptoms, especially in those critically ill ones with severe diseases (14). However, it is well documented that microbial cultures have their own limitations in this regard. They are usually time-consuming, and may cause misdiagnosis if the pathogenic bacterial/fungal burden is low or requires demanding culture conditions (16, 17). Serological diagnostic methods and ELISA-based diagnostic tests for antigen detections are expected to provide results in several hours and offer promising alternatives to microscopic and/or microbiological diagnosis. However, before these approaches can be widely adopted, several issues remain to be addressed, including adverse drug effects, improving the accuracy, and lowering the costs (18–20).

PCR-based molecular methods for pathogen detections are relatively simple and provide improved sensitivity compared to the microscopy, culture and serological methods, especially for samples with negative culture results and slow growth of pathogenic bacteria (21). PCR-based assays have various advantages over the other traditional methods, highly specific and sensitive. Several studies demonstrated the improved diagnostic yield of molecular examinations for pneumonia pathogens (22–24). However, little information is available regarding comparisons of pathogen detection techniques using BALF samples especially from critically ill subjects, including not only traditional microscopy and culture methods but also PCR-based molecular means. In this study, we employed PCR-based approaches for bacterial and fungal pathogen detections with BALF specimens in severe patients with pneumonia, and compared the molecular method with the traditional microscopic examinations, cultures, as well as antigenic marker-based serological assays.

## Materials and methods

### Patient enrollment and sample collection

This study enrolled critically ill patients who were admitted to the Intensive Care Unit (ICU) or Department of Pulmonary and Critical Care Medicine in a tertiary care hospital of Anhui Province in China between November 2020 and March 2021. Cases of interest were screened out with clinical and radiological evidence of pneumonia through daily surveillance of all BALF samples received by our clinical laboratory. Final inclusion criteria for the study were determined by our radiologists or consultant respiratory physicians according to the international guidelines for the management of adults with hospital-acquired, ventilator-associated, and healthcare-associated pneumonia, and for the invasive fungal diseases (25–27), and also based on the Chinese expert consensus statement issued by the Chinese Medical Association (28, 29).

BALF collection was strictly guided by the American Thoracic Society Clinical Practice Guideline (30). Briefly, BAL was performed through a fiberoptic bronchoscope in a wedge position within the selected bronchopulmonary segment. The total instilled volume was 100–150 ml (5–7 times of 20 ml of 0.9% sodium chloride solution). Post lavaging, the total volume (pooled aliquots) retrieved should be greater than or equal to 30% of the total instilled volume (30). Totally, 10–20 ml of BALF was harvested for pathogen identification. The collected samples were divided into two parts, one for incubation and microscopic examinations, and the other for storage at −20°C until DNA extraction was performed.

### Microscopic examinations

One milliliter of BALF specimen was centrifuged at 2,000 *g* for 10 min. The supernatant was discarded, the sediment was mixed thoroughly and aspirated with a sterile pipette. A drop of the mixture was evenly placed onto a clean slide. Two slides were prepared and subjected to Gram staining (BioMérieux, France) with double check (31).

### Cultures

Microbial cultures were performed according to routine clinical protocols. BALF was incubated on blood agar, chocolate agar, MacConkey, anaerobic blood agar, Sabouraud medium, potato dextrose agar, and/or *Candida* chromogenic plates for 48 h or more (32). Microbial strains were identified by matrix-assisted laser desorption ionization time-of-flight mass spectrometry (MALDI-TOF MS). The mass spectra were obtained *via* the platform of Vitek MS (bioMérieux, Marcy l’Étoile, France) with 99.9% confidence (33).

### GM and (1,3)-β-D-glucan (BDG) testing

GM concentrations of BALF were determined routinely by the Platelia EIA (Bio-Rad Laboratories) in our clinical laboratory. The optical density (OD) index cutoff of 1.0 was applied to the evaluation of diagnostic performance for the GM tests (34). The BDG tests with BALF were carried out according to the manufacturer’s instructions. The average rate of OD change for all points between 0 and 40 min was interpolated with the results of standard curve to determine the amount of BDG (35). Negative and positive controls were included in each run of the assays.

### DNA isolation, PCR, and sequencing

Total DNA was extracted from BALF using a QIAamp DNA Mini Kit (Qiagen, Hilden, Germany) according to the manufacturer’s protocol. Detection of bacteria was performed by targeting 16S rRNA with PCR. The primers for the 16S rRNA region were: 27F 5’-AGAGTTTGATCCTGGCTCAG-3′ and 1492R 5’-TACGGCTACCTTGTACGACTT-3′ (36). The optimized PCR conditions consisted of an initial denaturation at 94°C for 3 min, 30 cycles of denaturation at 94°C for 30 s, annealing at 56°C for 30 s, and extension at 72°C for 1.5 min, and a final extension at 72°C for 20 min. Pan-fungal primers for PCR detection of fungi included 18SF1 5’-AGAGTTTGATCCTGGCTCAG-3′ and 58SR1 5’-TTCGCTGCGTTCTTCATCGA-3′ (37); its optimal parameters for PCR were: one initial denaturation at 94°C for 5 min, followed by 35 cycles of denaturation at 94°C for 1 min, annealing at 47°C for 15 s and extension at 72°C for 20 s, and one last extension at 72°C for 10 min. The PCR products were semi-quantified by agarose gel electrophoresis and used as templates for sequencing (Sangon Biotech Co., Ltd., Shanghai, China). Sequencing data were analyzed in GenBank by BLAST. The PCR and subsequent sequencing and BLAST were referred to as the PCR based methods herein.

### Determination of limit of detection of cultures and PCR

Firstly, bacterial strains identified in this study were inoculated on blood agar plates and incubated at 37°C for 48 h. In the meantime, the fungal species involved were inoculated in Sabouraud medium and incubated at 28°C and/or 37°C for 7 d. Then, their culture colony suspensions were separately prepared with a turbidity of 0.5 (1 × 10^7^ CFU/ml). Each configured bacterial/fungal suspension was serially diluted 10 times in saline and split into two parts, of which one was for the LOD analysis of cultures and the other for that of PCR.

### Statistical analysis

Data were collected and subsequently analyzed on IBM SPSS Statistics 21.0 (SPSS Inc., Chicago, IL, USA), and *p* < 0.05 was considered significantly different. The sensitivity/specificity of the detecting methods were assessed according to the formulas of TP (true positive)/[TP + FN (false negative)] × 100% and TN (true negative)/[TN + FP (false positive)] × 100%, respectively. Positive predictive value (PPV) and negative predictive value (NPV) were calculated based on PPV = TP/(TP + FP) × 100% and NPV = TN/(TN + FN) × 100%, respectively. All statistics were reported as absolute values of their 95% confidence intervals (CIs).

## Results

### LOD of microbial culture and PCR-based methods

**Figure 1:**
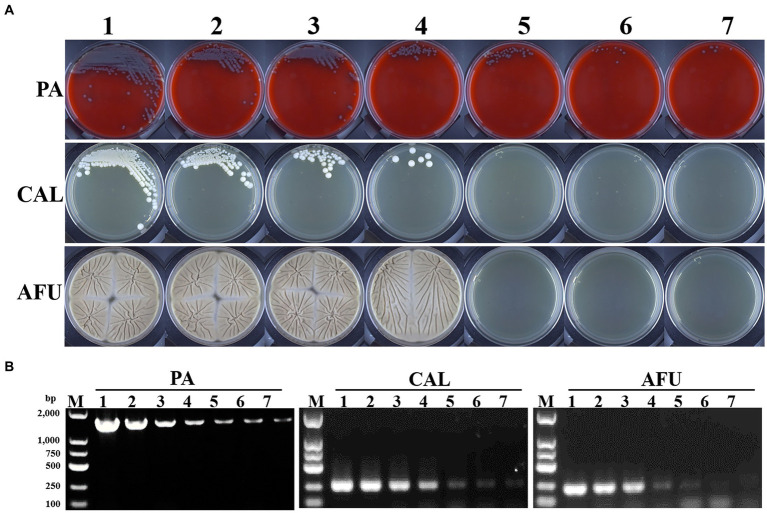
Limit of detection (LOD) of microbial culture and PCR-based methods. **(A)** Growth results of *Pseudomonas aeruginosa*, *Candida albicans* and *Aspergillus fumigatus* with different turbidity levels on blood agar or Sabouraud medium plates, respectively. **(B)** Electropherograms of *Pseudomonas aeruginosa*, *Candida albicans* and *Aspergillus fumigatus* with different turbidity levels. PA, *Pseudomonas aeruginosa*; CAL, *Candida albicans*; AFU, *Aspergillus fumigatus*. 1–7: turbidity of bacterial/fungal solutions of 10^6^, 10^5^, 10^4^, 10^3^, 10^2^, 10^1^, 1 CFU/ml, respectively.

Typically to establish the LOD in the present study, *Pseudomonas aeruginosa* was used for bacterial detection, and *Candida albicans* and *Aspergillus fumigatus* for fungal identification. Briefly, the LOD of bacteria was determined to be less than 1 CFU/ml for both cultures (Figure 1A) and PCR (Figure 1B). *Candida albicans* and *Aspergillus fumigatus* showed similar LOD by cultures (10^3^ CFU/ml). While, PCR based diagnostic methods showed improved detection performance with LOD of 1 CFU/ml for *Candida albicans* and 10 CFU/ml for *Aspergillus fumigatus*, respectively (Figures 1A,B).

### Comparison of microscopy, culture, antigenic marker, and PCR-based methods

During the study period, a total of 342 BALF samples were analyzed. The results from the microscopy, culture and PCR-based assays were listed in Table 1 (PCR-based method as the criterion standard). For overall bacterial and fungal detections, the microscopy showed the lowest detection rate, with only 29 (8%) samples presenting bacterial and/or fungal readout. The cultures displayed a slightly higher sensitivity (33% sensitivity; 95% CI, 26 to 41%) than the microscopy (18% sensitivity; 95% CI, 13 to 25%). The PPV and NPV were 100 and 58% for microscopy diagnosis, and 100 and 63% for cultures, respectively.

**Table 1:** Comparison of microscopy and culture methods with PCR-based assay as criterion standard.

	Methods and results	PCR*			Sensitivity (%) (95% CI)	Specificity (%) (95% CI)	PPV^†^ (%)	NPV^#^ (%)
		Positive	Negative	Total				
*Overall* *^$^*	*Microscopy*							
	Positive	29	0	29	18 (13–25)	100 (97–100)	100	58
	Negative	131	182	313				
	Total	160	182	342				
	*Culture*							
	Positive	53	0	53	33 (26–41)	100 (97–100)	100	63
	Negative	107	182	289				
	Total	160	182	342				
*Bacteria*	*Microscopy*							
	Positive	25	0	25	19 (13–27)	100 (98–100)	100	67
	Negative	106	211	317				
	Total	131	211	342				
	*Culture*							
	Positive	31	0	31	24 (17–32)	100 (98–100)	100	68
	Negative	100	211	311				
	Total	131	211	342				
*Fungi*	*Microscopy*							
	Positive	6	0	6	12 (5–24)	100 (98–100)	100	86
	Negative	46	290	336				
	Total	52	290	342				
	*Culture*							
	Positive	27	0	27	52 (38–66)	100 (98–100)	100	92
	Negative	25	290	315				
	Total	52	290	342				
*Candida* spp.	*Microscopy*							
	Positive	4	0	4	24 (8–50)	100 (99–100)	100	96
	Negative	13	325	338				
	Total	17	325	342				
	*Culture*							
	Positive	13	0	13	76 (50–92)	100 (99–100)	100	99
	Negative	4	325	329				
	Total	17	325	342				
*Aspergillus* spp.	*Microscopy*							
	Positive	2	0	2	17 (3–49)	100 (97–100)	100	94
	Negative	10	150	160				
	Total	12	150	162				
	*Culture*							
	Positive	9	0	9	75 (43–93)	100 (97–100)	100	98
	Negative	3	150	153				
	Total	12	150	162				
	*BDG test*							
	Positive	5	27	32	42 (16–71)	82 (75–88)	16	95
	Negative	7	123	130				
	Total	12	150	162				
	*GM test*							
	Positive	11	46	57	92 (60–100)	69 (61–76)	19	99
	Negative	1	104	105				
	Total	12	150	162				

*Detection method as gold standard.

† Positive predictive value (PPV) = true positive (TP)/(TP + false positive [FP]) × 100%.

# Negative predictive value (NPV) = true negative (TN)/(TN + false negative [FN]) × 100%.

$ Here it included detections of all the bacteria and fungi together.

As for bacterial detections, the microscopy also had a lower sensitivity (19% sensitivity; 95% CI, 13 to 27%) than cultures (24% sensitivity; 95% CI, 17 to 32%). The PPV and NPV for microscopy diagnosis were 100 and 67%, and for cultures 100 and 68%, respectively (Table 1).

According to our data, the cultures displayed a much higher sensitivity (52% sensitivity; 95% CI, 38 to 66%) for fungal detections when compared with the microscopy (12% sensitivity; 95% CI, 5 to 24%). The PPV and NPV were 100 and 86% for microscopy diagnosis, and 100 and 92% for cultures, respectively. We further compared the microscopy, culture, antigenic marker-based approaches including GM and BDG tests, as well as PCR-based assays for *Aspergillus* spp. and/or *Candida* spp. Results revealed that cultures had a high sensitivity of detection for *Candida* spp. (76% sensitivity; 95% CI, 50 to 92%) and *Aspergillus* spp. (75% sensitivity; 95% CI, 43 to 93%). Notably, GM tests exhibited the highest sensitivity (92% sensitivity; 95% CI, 60 to 100%) and the lowest specificity (69% specificity; 95% CI, 61 to 76%) for *Aspergillus* spp. The other approaches showed poor detection sensitivities for *Candida* spp. and *Aspergillus* spp. (Table 1).

There was poor agreement among traditional microscopy, culture and PCR assays for bacterial detections (Table 2; kappa value, 0.184 to 0.277). However, traditional cultures were moderately consistent with the PCR-based method for fungal species detections, particularly for identification of *Candida* spp. and *Aspergillus* spp., with kappa values of 0.861 and 0.847, respectively.

**Table 2:** Kappa coefficients of diagnostic tests for BAP/FAP.

	Microscopy	Culture	GM^*^	BDG test^†^
*Total*				
*Microscopy*				
*Culture*	0.206			
*PCR*	0.191	0.345		
*BAP*				
*Microscopy*				
*Culture*	0.184			
*PCR*	0.225	0.277		
*FAP*				
*Microscopy*				
*Culture*	0.345			
*PCR*	0.181	0.647		
*Candida* spp.				
*Microscopy*				
*Culture*	0.460			
*PCR*	0.369	0.861		
*Aspergillus* spp.				
*Microscopy*			0.045	0.097
*Culture*	0.351		0.196	0.065
*PCR*	0.270	0.847	0.224	0.134
*BDG test*			0.082	

*, † GM and BDG were only involved in the calculation in the *Aspergillus* spp. test.

The present study revealed that PCR-based method demonstrated a higher sensitivity than that of the microscopy or cultures. The detailed data of bacterial and fungal species detected by microscopic examination, culture and PCR-based methods were summarized in Tables 3, 4. Bacterium-associated pneumonia was mainly caused by Gram-negative bacteria. Among them, *Pseudomonas* spp., *Burkholderia* spp. and *Klebsiella* spp. were the major pathogens. It was worth noting that nearly one third of infections were caused by fungi, particularly *Candida* spp. and *Aspergillus* spp. In addition, PCR-based method was also able to detect certain pathogenic bacteria that failed the conventional methods, such as *Prevotella* spp., *Cladosporium* spp., *Malassezia* spp., etc. (Tables 3, 4). Moreover, one sample with mixed infections was detected by PCR-based assays.

**Table 3:** Detailed comparison of microscopy, cultures, and PCR for BAP detection and species identification.

Bacteria	Species	Genus	Microscopy	Culture	PCR
*Total*			25/342	31^*^/342	131/342
*Gram-negative bacteria*			16	33	81
	*Pseudomonas* spp.		2	10	18
		*P. aeruginosa*	2	10	17
		*P. fragi*	0	0	1
	*Burkholderia* spp.	*B. contaminans*	1	2	15
	*Klebsiella* spp.		7	12	17
		*K. pneumoniae*	7	9	14
		*K. oxytoca*	0	3	3
	*Alcaligenes* spp.		1	0	8
	*Acinetobacter* spp.		2	7	7
		*A. baumannii*	2	7	6
		*A. ursingii*	0	0	1
	*Prevotella* spp.		0	0	4
	*Chryseobacterium* spp.		0	0	4
		*C. indologenes*	0	0	3
		*C. cucumeris*	0	0	1
	*Stenotrophomonas* spp.	*S. maltophilia*	0	1	2
	*Bacteroides* spp.	*B. zoogleoform*	3	0	3
	*Achromobacter* spp.	*A. denitrificans*	0	1	1
	*Neisseria* spp.	*N. mucosa*	0	0	1
	*Photobacterium* spp.		0	0	1
*Gram-positive bacteria*			9	2	50
	*Bacillus* spp.		5	0	23
		*B. cereus*	1	0	11
		*P. polymyxa*	4	0	11
		*B. subtilis*	0	0	1
	*Streptococcus* spp.		2	0	12
		*S. oralis*	1	0	3
		*S. pneumoniae*	0	0	2
		*S. parasanguinis*	0	0	2
		*S. pseudopneumoniae*	0	0	3
		*S. rubneri*	0	0	1
		*S. mitis*	1	0	1
	*Granulicatella* spp.	*G. adiacens*	1	0	6
	*Staphylococcus* spp.		0	2	4
		*S. epidermidis*	0	2	2
		*S. aureus*	0	0	2
	*Abiotrophia* spp.	*A. defectiva*	0	0	2
	*Rothia* spp.	*R. mucilaginosa*	1	0	1
	*Gemella* spp.	*G. morbillorum*	0	0	2

*A total of 31 samples were cultured with bacteria, while there were cases where multiple bacteria were identified from the same sample cultures.

**Table 4:** Detailed comparison of microscopy, cultures, and PCR for FAP detection and species identification.

Fungi	Species	Genus	Microscopy	Culture	PCR
*Total*			6/342	27/342	52^*^/342
	*Candida* spp.		4	13	17
		*C. albicans*	4	10	13
		*C. tropicalis*	0	2	2
		*C. parapsilosis*	0	1	1
		*S. cerevisiae*	0	0	1
	*Aspergillus* spp.		2	11	18
		*A. fumigatus*	2	10	14
		*A. sydowii*	0	0	2
		*A. flavus*	0	1	1
		*A. versicolor*	0	0	1
	*Cladosporium* spp.		0	0	6
		*C. oxysporum*	0	0	3
		*C. cladosporioides*	0	0	2
		*C. aciculare*	0	0	1
	*Malassezia* spp.		0	0	4
		*M. globosa*	0	0	2
		*M. restricta*	0	0	2
	*Penicillium* spp.		0	2	4
		*P. oxalium*	0	2	2
		*P. corylophilum*	0	0	1
		*P. chrysogenum*	0	0	1
	Others	*Cryptococcus neoformans*	0	1	1
		*Peronospora* spp.	0	0	1
		*Rhizopus microsporus* var. *rhizopodiformis*	0	0	2

*A total of 52 samples were identified by PCR for fungus strains, while there was one case with multiple strains identified.

### Patient characteristics

In the present study, we observed the male to female ratios in the groups with or without BAP/FAP were 1.81 and 1.44, respectively. In addition, it was demonstrated that the age was positively correlated with the incidence of BAP/FAP. As summarized in Table 5, the most susceptible age was over 50 years old among patients with BAP/FAP, accounting for 68%. Comparatively, significant difference was shown between patients with BAP/FAP and those without in the age group of over 65 years old (*p* < 0.05). Moreover, the length of hospital stay was positively correlated with the rate of BAP/FAP. Almost half of the infections occurred in patients hospitalized for more than 14 days (43%). Importantly, among the various relevant risk factors which substantially influence the BAP/FAP incidence, we found that catheters, tracheal intubation, ventilator support, arteriovenous cannulation, tracheotomy and cerebral diseases were statistically different between groups (*p* < 0.05, Table 5). Whereas, the remaining risk factors we observed did not show significant differences (*p* > 0.05), including surgery, hemodialysis/peritoneal dialysis, immunosuppressive treatment, hypertension, diabetes, heart diseases, chronic liver diseases, chronic kidney diseases, neoplastic diseases and organ transplantation. When compared with patients with BAP/FAP, the outcomes of the ones without such infections were significantly improved (*p* < 0.05).

**Table 5:** Demographic and clinical characteristics of patients.

Characteristics	Patients with BAP/FAP (%)	Patients without BAP/FAP (%)	*P*
Gender			
Male	64	59	0.360
Female	36	41	
Age (years)			
0–14	10	16	0.131
15–49	22	21	0.818
50–65	29	37	0.157
>65	39	26	<0.05
Hospital stay (days)			
0–7	18	21	0.579
8–14	39	48	0.120
≥15	43	31	<0.05
Risk factors			
*Invasive intervention/procedure*			
Catheters	17	7	<0.05
Tracheal intubation	20	9	<0.05
Ventilator support	18	8	<0.05
Arteriovenous cannulation	17	7	<0.05
Tracheotomy	9	1	<0.05
Surgery	42	38	0.474
*Immunosuppressive state*			
Immunosuppressive treatment	14	13	0.779
*Underlying disease*			
Hypertension	17	13	0.418
Cerebral disease	13	2	<0.05
Chronic liver disease	6	7	0.766
Chronic kidney disease	8	5	0.349
Neoplastic disease	5	6	0.926
Transplant	3	1	0.602
Outcomes			
Improvement	77	85	<0.05
Failure	0	1	
Death	2	0	
Others	21	14	

## Discussion

Pneumonia is the leading cause of hospitalization and death worldwide. According to the 2019 data from Global Burden of Diseases (GBD), lower respiratory tract infections (LRTIs) including pneumonia and bronchiolitis, affected 489 million people globally (15). Hospital-acquired pneumonia (HAP) and VAP are the most common hospital-acquired infections in China. The specificity of patients’ clinical manifestations, laboratory tests and imaging diagnosis is relatively low, and its treatment is difficult due to the presence of drug-resistant microorganisms, resulting in prolonged hospitalization and a significantly higher mortality rate, which increases the medical and economic burden on the country and families (28). Therefore, rapid and accurate diagnostic methods are urgently needed.

Bronchoscopy allows direct access to the lower airways and direct sampling of bronchial and parenchymal tissues at the site of inflammation in the lungs. Thus, it suggests that BALF may provide useful information for the diagnosis of various lung diseases, including BAP/FAP. As a matter of fact, microscopic examinations of clinical specimens can be initial, cost-effective tests that are able to rapidly identify many invasive bacterial/fungal infections. They use relatively inexpensive materials, do not require highly specialized equipment, and results can often be obtained within a few hours post specimen collection. For the detection of fungi in particular, the visibility of fungi in clinical specimens can be further enhanced by the addition of Calcofluor white, a fluorescent moiety that binds to the chitin of fungal cell wall, or Lactophenol cotton blue which stains the outer cell wall. Other stains are often utilized for microscopic examinations, such as India ink wet mount, which helps to visualize encrusted fungi, particularly *Cryptococcus neoformans*. Although a negative microscopic examination cannot rule out infectious diseases, visualization of pathogens in specimens is frequently able to give preliminary information to facilitate the selection of empirical treatments. However, under conventional laboratory conditions, it is difficult to achieve high detection sensitivities and distinguish mixed infections. In this study, merely 18% of PCR positive samples were detected by the microscopy. In addition, only 9% of mixed infection cases were determined in the present research. This phenomenon was also observed in other studies (38).

Microbial cultures remain the key methods for diagnosing bacterial/fungal pulmonary infections. Since they are more sensitive than microscopic examinations, cultures should be performed on all qualified clinical specimens especially when assessing suspected infectious diseases, regardless of whether pathogens are visible by microscopic examinations. The preparation of clinical specimens is critical to the success of bacterial/fungal cultures. Generally, for respiratory samples, the purulent portion should be inoculated directly onto a suitable culture medium. In addition, many pathogens are difficult to culture despite the application of specialized medium materials with appropriate handling of specimens. In this study, 33% of PCR-confirmed positive samples were detected positive by the culture-based method. The reasons for culture failure may be mainly due to the demanding environmental and nutrient requirements of pathogens and/or the sensitivity limits of the methodology itself. The phenomenon has also been observed in other studies (39–42).

GM test is a polysaccharide component of cell wall of *Aspergillus* that can be measured in sera and other body fluids to evaluate or as evidence of invasive aspergillosis. In an attempt to improve the diagnostic yield for invasive pulmonary aspergillosis, it has focused on directly testing BALF for the presence of GM. In recent years, several studies have shown that the sensitivity of BALF GM assays was higher than that of serum ones for identification of *Aspergillus* associated with pulmonary aspergilloma (43). In this study, we also observed a high sensitivity of the BALF GM tests. This might involve locally invasive diseases allowing the leakage of GM. Therefore, using BALF rather than blood samples for GM testing would help to improve the detection rate of invasive pulmonary aspergillosis. The detection of serum BDG, a component of fungal cell wall, is another useful tool in diagnosing invasive fungal infections. Some studies have reported that the accuracy of BDG tests in BALF was marginal (44–46). In this study, only 42% of PCR-confirmed positive fungal samples were identified to be positive by BDG tests. Thus, the BDG results of BALF should not be interpreted alone, but ought to be used in combination with clinical features, image findings and other laboratory results for the diagnosis of invasive fungal diseases.

In this study, PCR-based method was used as the reference standard because of its high sensitivity and specificity as well as advantages over the microscopy and culture-based methods, particularly in cases with low-level bacterial/fungal colonies. Here, we evaluated the PCR-based method for detecting the pathogens of clinically indicated bacterial/fungal pneumonia by BALF. It was interesting to observe that bacterial and fungal universal primer-based PCR method showed an excellent detection sensitivity. Our results suggested that PCR combined with sequencing might be highly suitable for pathogen detections in BALF samples from patients with suspected bacterial/fungal pneumonia.

Although Gram-negative (GN) bacteria are responsible for the majority of HAP/VAP, an increasing number of fungal infections can currently be identified and responsible for many infections, particularly *Candida* and *Aspergillus species* (47–50). Similarly, our data revealed that GN bacteria were still the principal pathogens causing pulmonary infections, while fungi were identified and responsible for almost 33% of the BAP/FAP in this study.

Various risk factors, alone or in combination, influence the incidence of BAP/FAP, including age, length of hospital stay, underlying diseases, etc. (26). In our study, the patients with BAP/FAP were nearly older adults and experienced relatively long length of hospital stay, nearly half were over 50 years old and with >14 days of hospital stay. Amidst the indicated risk factors, catheters, tracheal intubation, ventilator support, arteriovenous cannulation, tracheotomy and cerebral diseases were highly indicative of the occurrence of BAP/FAP in the present study. In addition, our findings illustrated that the patients with BAP/FAP had significantly worse clinical outcomes compared to those without.

In this investigation, we confirmed that BALF was likely to provide useful information for the diagnosis of BAP/FAP. Standard microscopic examinations and cultures performed relatively poor, probably because our samples had very low bacterial and/or fungal densities. The present research demonstrated that combined PCR-based strategies were likely able to be practical and effective surveillance tools for diagnosis of BAP/FAP. Moreover, our analysis indicated that the age, length of hospital stay, invasive procedures and cerebral diseases should be considered as the main risk factors for BAP/FAP. Thus, screening for BALF in patients with suspected pulmonary infections in the presence of these risk elements using the combined diagnostic methods is expected to facilitate the early diagnosis of pulmonary infections and to improve the prognosis of patients.

## Data availability statement

The datasets presented in this study can be found in online repositories. The names of the repository/repositories and accession number(s) can be found at: https://www.ncbi.nlm.nih.gov/ & OQ716403, OQ716460, OQ716462, OQ716465, OQ719734, OQ719745, OQ719749, OQ716554, OQ716562, OQ716574, OQ716582, OQ716675, OQ716681, OQ716706, OQ716705, OQ716708, OQ719722, OQ719733, OQ716709, OQ716711, OQ718977, OQ718976, OQ718978, OQ718979, OQ718981, OQ718982, OQ718983, OQ718984, OQ719023, OQ719024, OQ719026, OQ719027, OQ723446, OQ719032, OQ719033, OQ723442, OQ723444, OQ723445, OQ723440, OQ723441, OQ723443, OQ723447, OQ723448, OQ723449, OQ723450, OQ723455, OQ723452, OQ723453, OQ723457, OQ719617.

## Ethics statement

The studies involving human participants were reviewed and approved by the Life Ethics Committee of Anhui Medical University. The patients/participants provided their written informed consent to participate in this study.

## Author contributions

BW, JX, LZ, and FL conceived and designed the experiments and contributed to the interpretation of results and assisted in writing the manuscript. BW, LZ, YW, JJ, FL, YX, YH, and MZ designed the research protocol and performed the experiments. JX, YW, JJ, YX, YH, and MZ performed data acquisition and analysis. All authors contributed to the article and approved the submitted version.

## Funding

This study was supported by the National Natural Science Foundation of China (81601446) (BW), the Natural Science Foundation of Anhui Province (1708085QH210) (BW), and the key clinical specialty project of Anhui Province (3101005004160) (YX). The funders had no role in the study design, data collection, and analysis, decision to publish, or preparation of the manuscript.

## Conflict of interest

The authors declare that the research was conducted in the absence of any commercial or financial relationships that could be construed as a potential conflict of interest.

## Publisher’s note

All claims expressed in this article are solely those of the authors and do not necessarily represent those of their affiliated organizations, or those of the publisher, the editors and the reviewers. Any product that may be evaluated in this article, or claim that may be made by its manufacturer, is not guaranteed or endorsed by the publisher.
